# microRNA-200c regulates KLOTHO expression in human kidney cells under oxidative stress

**DOI:** 10.1371/journal.pone.0218468

**Published:** 2019-06-14

**Authors:** Kenichi Morii, Satoshi Yamasaki, Shigehiro Doi, Taisuke Irifuku, Kensuke Sasaki, Toshiki Doi, Ayumu Nakashima, Koji Arihiro, Takao Masaki

**Affiliations:** 1 Department of Nephrology, Hiroshima University Hospital, Hiroshima, Japan; 2 Center for Rheumatic Diseases, Kurume University Medical Center, Kurume, Japan; 3 Department of Stem Cell Biology and Medicine, Graduate School of Biomedical & Health Sciences, Hiroshima University, Hiroshima, Japan; 4 Department of Anatomical Pathology, Hiroshima University Hospital, Hiroshima, Japan; National Institutes of Health, UNITED STATES

## Abstract

KLOTHO deficiency is associated with the progression of kidney dysfunction, whereas its overexpression exerts renoprotective effects. Oxidative stress suppresses KLOTHO expression in renal epithelial cells but upregulates microRNA-200c (miR-200c) in human umbilical vein endothelial cells. In this study, we investigated whether oxidative stress-induced miR-200c is implicated in KLOTHO downregulation in human renal tubular epithelium (HK-2) cells. HK-2 cells were stimulated with hydrogen peroxide (H_2_O_2_) to examine the effect of oxidative stress. A luciferase reporter containing the *KLOTHO* 3′-UTR was used to investigate the effect of miR-200c on *KLOTHO* mRNA metabolism. The expressions of KLOTHO, oxidative stress markers, and miR-200c were determined in human kidney biopsy specimens. H_2_O_2_ suppressed KLOTHO expression without a reduction in *KLOTHO* mRNA levels but upregulated miR-200c expression. Similarly, transfection of a miR-200c mimic reduced KLOTHO levels and luciferase activity without a reduction in *KLOTHO* mRNA levels. In contrast, transfection of a miR-200c inhibitor maintained KLOTHO expression. Immunofluorescent assay revealed KLOTHO was present in the cytosol and nuclei of HK-2 cells. In human kidney biopsies, KLOTHO expression was inversely correlated with levels of oxidative stress markers (8-hydroxy-2′-deoxyguanosine: ρ = −0.38, *P* = 0.026; 4-hydroxy-2-hexenal: ρ = −0.35, *P* = 0.038) and miR-200c (ρ = −0.34, *P* = 0.043). Oxidative stress-induced miR-200c binds to the *KLOTHO* mRNA 3′-UTR, resulting in reduced KLOTHO expression.

## Introduction

Chronic kidney disease (CKD) is recognized as a risk factor in the development of end-stage kidney disease [1], and all-cause mortality [2–5]. Consequently CKD has a substantial economic burden [6]. Currently, oxidative stress is defined as an imbalance between the production of reactive oxygen species (ROS) and anti-oxidant defenses [7]. Although past studies have reported that increased ROS levels play a pivotal role in the progression of CKD [8,9], ROS are also involved in physiological processes, including cell signaling [10], gene expression [11], and cell growth [12]. Therefore, inhibition of ROS has not been established as a therapy for CKD [13]. In addition to ROS damage *per se*, recent studies have revealed that oxidative stress also participates in renal damage through the downregulation of renoprotective factors [14–16]. These findings indicate that oxidative stress-induced downregulation of such factors is a potential therapeutic strategy to prevent the progression of CKD.

KLOTHO is a single-pass transmembrane protein consisting of 1012 amino acids [17,18], and is strongly and weakly expressed in distal renal tubular epithelial cells and proximal renal tubular epithelial cells, respectively [19]. In addition to phosphate excretion, KLOTHO exhibits multiple functions, including the amelioration of oxidative stress [20,21], and inhibition of signaling pathways of insulin growth factor [22], Wnt/β-catenin [23], transforming growth factor-β1 [24], and mechanistic target of rapamycin signaling [25]. Overexpression of the *Klotho* gene or injection of KLOTHO protein shows beneficial effects in rodent models of various renal diseases [26]. These findings suggest that maintaining KLOTHO expression is a novel therapeutic strategy during the development of CKD. However, another study showed that hydrogen peroxide (H_2_O_2_), a ROS, contributed to the downregulation of KLOTHO expression in renal epithelial cells [14,15], causing renal damage [27]. Therefore, the underlying mechanism by which H_2_O_2_ decreases KLOTHO expression should be clarified to identify a therapeutic target.

Gene expression is regulated by epigenetic alterations, including histone modification, DNA methylation and microRNA (miRNA) expression [28–31]. Among these, miRNAs, which are small, endogenous, non-coding and single-stranded RNAs of 21–25 nucleotides, play a major role in repressing gene expression post-transcriptionally by binding to specific sites within the 3′-untranslated region (3′-UTR) of a target gene mRNA [32–34]. H_2_O_2_ upregulated microRNA-200c (miR-200c) in human umbilical vein endothelial cells [35], and, notably, there are two putative miR-200c binding sites in the 3′-UTR of the *KLOTHO* mRNA. These findings led us to the hypothesis that H_2_O_2_ suppresses KLOTHO expression through the induction of miR-200c. To test this, we investigated whether miR-200c regulates KLOTHO expression in kidney cells under oxidative stress.

In this study, we show that H_2_O_2_ suppresses KLOTHO expression without reducing levels of *KLOTHO* mRNA. We also show that H_2_O_2_-induced miR-200c downregulates KLOTHO expression by binding to the *KLOTHO* mRNA 3′-UTR. Last, KLOTHO expression is associated with markers of oxidative stress and miR-200c in renal biopsy samples from IgA nephropathy patients. These findings indicate that oxidative stress suppresses KLOTHO expression through the induction of miR-200c.

## Materials and methods

### Cell culture

Human renal proximal tubular epithelium (HK-2) cells were obtained from the American Type Culture Collection (CRL-2190, Lot No. 61218770, Manassas, VA). Mycoplasma was not detected during the experimental period. The cells were maintained in RPMI-1640 medium containing 10% fetal bovine serum (FBS) (Nichirei Bio Science, Tokyo, Japan) and penicillin/streptomycin (Nacalai, Kyoto, Japan). For stimulations, HK-2 cells were treated with 100 μM H_2_O_2_ (Sigma-Aldrich, St. Louis, MO) for 6–24 hours (hrs) and 100–1000 μM paraquat (Sigma-Aldrich) for 24 hrs. ERK (#6560), JNK (#6232), p38 (#6564) and control (#6568) siRNAs were purchased from Cell Signaling Technology (Danvers, MA). Cells were transfected using Lipofectamine 2000 Reagent (Invitrogen, Waltham, MA) in accordance with the manufacturer’s protocol. After incubation with transfection complexes for 24 hrs, the medium was changed, and the cells were stimulated with 100 μM H_2_O_2_ for 24 hrs.

### miRNA transfection

To examine the effect of miR-200c in HK-2 cells, hsa-miR-200c mimic (miRVana miRNA mimic, Applied Biosystems, Foster City, CA) or mimic control (miRVana miRNA mimic negative control, Applied Biosystems) were transfected into HK-2 cells using Lipofectamine RNAiMAX (Invitrogen) in accordance with the manufacturer’s instructions. To evaluate the inhibitory effects of miR-200c and miR-21 on KLOTHO expression, hsa-miR-200c and hsa-miR-21 inhibitor (miRVana miRNA inhibitor, Applied Biosystems) or inhibitor control (miRVana miRNA inhibitor negative control, Applied Biosystems) were transfected into HK-2 cells using Lipofectamine RNAiMAX (Invitrogen). Mimic control or inhibitor control were used as negative controls.

### Western blotting

Western blot analysis was performed as described previously [24,36,37]. Primary antibodies used were rat monoclonal anti-human KLOTHO antibody (KM2076, TransGenic, Kobe, Japan), mouse monoclonal anti-α-TUBULIN (TUBA) antibody (T9026, Sigma-Aldrich, St. Louis, MO), rabbit monoclonal anti-ERK1/2 antibody (#4696, Cell Signaling Technology), rabbit monoclonal anti-JNK antibody (#9252, Cell Signaling Technology) and rabbit monoclonal anti-p38 antibody (#8690, Cell Signaling Technology). The intensity of detected proteins was determined using ImageJ software (version 1.50i; National Institutes of Health, Bethesda, MD).

### Gene expression

#### 1) Quantitative PCR (q-PCR) for *KLOTHO*, *ACTIN B* (*ACTB*), pri-hsa-miR-200c and pri-hsa-miR-21

Total RNA was extracted from conditioned cells using an RNeasy Mini Kit (Qiagen, Venlo, Netherlands). For the synthesis of complementary DNA (cDNA), total RNA was reverse transcribed using a High-Capacity cDNA Reverse Transcription Kit (Applied Biosystems). *KLOTHO* and *ACTB* mRNAs were quantified using TaqMan Gene Expression Assays (assay ID: Hs00183100_ml for *KLOTHO* and assay ID: Hs01060665_gl for *ACTB*) (Applied Biosystems) and a 7500 Fast Real-Time PCR (RT-PCR) System (Applied Biosystems). *ACTB* was used to verify equal sample loading. The expressions of pri-hsa-miR-200c and pri-hsa-miR-21 were quantified by TaqMan Pri-miRNA Assays (assay ID: Hs03303157_pri for pri-hsa-miR-200c, Hs03302625_pri for pri-hsa-miR-21) (Applied Biosystems) and a 7500 Fast RT-PCR System. The amplification of specific PCR products was confirmed by the 2^(−ΔΔCT)^ method with dissociation curve analysis for each primer pair.

#### 2) q-PCR for miRNA

For q-PCR analysis of hsa-miR-200c, hsa-miR-21 and U6 snRNA, RNA was extracted from conditioned cells using a miRNeasy Mini Kit (Qiagen). Five nanograms of RNA were converted to cDNA using a TaqMan MicroRNA Reverse Transcription Kit (Applied Biosystems). q-PCR was performed using TaqMan MicroRNA Assays and a 7500 Fast RT-PCR. U6 snRNA was used as a reference gene.

### *KLOTHO* 3*′*-UTR reporter assay

A *KLOTHO* 3′-UTR reporter clone in pMirTarget (pMirTarget-KL3′-UTR) was obtained from OriGene (SC217236, Rockville, MD). HK-2 cells were transfected with the plasmid for 4 hrs using Lipofectamine 2000 Reagent (Invitrogen) in accordance with the manufacturer′s protocol. Has-miR-200c mimic, mimic control, has-miR-200c inhibitor or inhibitor control were simultaneously transfected with the reporter plasmid in some experiments. After changing the medium, HK-2 cells were cultured for a further 12 hrs before sampling. The cells were lysed using passive lysis buffer (Promega, Madison, WI) and expression from the luciferase reporter construct was quantified using the Luciferase Reporter Assay System (Promega) on an Infinite 200Pro plate reader (Tecan, Kanagawa, Japan). The luciferase activity was normalized against protein quantity.

### Plasmid construction

Site-directed mutation of the miR-200c target sites in the pMirTarget-KL3′-UTR was generated using a QuikChange Lightning Site-Directed Mutagenesis Kit (Agilent Technologies, Santa Clara, CA) in accordance with the manufacturer’s instructions. The resulting plasmid was named pMirTarget-KL3′-UTR-MUT, which contained two 6 nucleotide substitutions at sites 568–573 and 1904–1909. Primer pairs used for construction were as follows; (forward, 5′-GAATGTTCCTTTCGAAAGCAATGCTTCTATCAAATACTCTGCGGAATTTATGTATCTGGTTAATGACATACTTGGAGAGCAA-3′; reverse, 5′-TTGCTCTCCAAGTATGTCATTAACCAGATACATAAATTCCGCAGAGTATTTGATAGAAGCATTGCTTTCGAAAGGAACATTC-3′) and (forward, 5′-TCCTTGACTGTAAAGAGAAGTAATTTTGCTCCTTGATAACTGCGGATATTAATAATAAATCTGCCTGCAACTTTTTGCCTTCTT-3′; reverse, 5′-AAGAAGGCAAAAAGTTGCAGGCAGATTTATTATTAATATCCGCAGTTATCAAGGAGCAAAATTACTTCTCTTTACAGTCAAGGA-3′).

### Clinical sample collection and ethics statement

Kidney specimens were obtained by renal biopsy at Hiroshima University Hospital between April 2014 and December 2016 from 35 patients who were diagnosed with IgA nephropathy. The patients’ demographic and clinical characteristics are shown in S1 Table. The Japanese glomerular filtration rate (GFR) equation based on serum creatinine (Cr) was used to estimate glomerular filtration rate (eGFR). eGFR (mL/min/1.73 m^2^) = 194 × Cr^−1.094^ × Age^−0.287^ (× 0.739 if female). This study adhered to the declaration of Helsinki and was approved by the Ethics Committee of Hiroshima University (E-633-2). Informed consent was obtained in the form of opt-out on the web-site. The ethics committee waived the requirement for written informed consent because of the retrospective nature of the study.

### Histology and immunohistochemistry of human kidney tissue

The following primary antibodies were used: rat monoclonal anti-human KLOTHO antibody (KM2076, TransGenic), mouse monoclonal anti-8-hydroxy-2'-deoxyguanosine (8-OHdG) antibody (MOG-020P, Japan Institute for the Control of Aging, Shizuoka, Japan) and mouse monoclonal anti-4-hydroxy-2-hexenal (4-HHE) antibody (MHH-030n, Japan Institute for the Control of Aging). Immunostaining of KLOTHO and 8-OHdG was performed as described previously [38]. 4-HHE was identified with the EnVision System (Dako, Santa Clara, CA). A positive area was quantified as the mean of five randomly selected fields using ImageJ software (National Institutes of Health).

### Immunofluorescence assay

HK-2 cells were washed with phosphate-buffered saline (PBS), fixed in 4% paraformaldehyde (Nacalai) and permeabilized with 0.5% Triton X-100 (Nacalai) at room temperature. After blocking with 5% Blocking One Histo (Nacalai) for 10 minutes (min), the cells were incubated with rabbit polyclonal KLOTHO antibody (1:100, PA5-21078, ThermoFisher Scientific) at 37°C for 30 min. After washing with PBS, the cells were incubated with Alexa Flour 488 goat anti-rabbit IgG (1:10,000, Invitrogen) at 37°C for 30 min in the dark. The nucleus was labeled with 4′,6-diamidino-2-phenylindole (DAPI) (H-1200, Vector Laboratories). Images were captured using a Keyence BZ-9000 fluorescence microscope.

### *In situ* hybridization

*In situ* hybridization (ISH) was performed on formalin-fixed paraffin-embedded human kidney biopsy specimens. Double digoxigenin (DIG)-labeled miRNA probes were designed by Exiqon (Venlo, Netherlands) to target has-miR-200c-3p. ISH was performed using a miRCURY LNA microRNA ISH Optimization Kit (Exiqon) in accordance with the manufacturer’s instructions. Proteinase-K incubation was performed with 15 μg/mL for 25 min. The miRNA probe was used at 80 nM, and the U6 snRNA probe at 2 nM. The U6 snRNA and scrambled probes were used as a positive technical control and a negative control, respectively. The miR-200c positive area was quantified as the mean of five randomly selected fields using LuminaVision (version 4.2.1.2; Mitani, Tokyo, Japan).

### Statistical analysis

Results are expressed as the mean ± standard deviation. Statistical analyses were performed using SPSS statistical software (version 25; IBM Corporation, Armonk, NY). Comparison between two groups was analyzed by the Mann-Whitney *U*-test. For multiple group comparison, the Mann-Whitney *U*-test with Bonferroni correction was applied. The correlation was calculated using Spearman’s rank correlation coefficient. Values of *P* < 0.05 were considered statistically significant.

## Results

### KLOTHO expression is inhibited by H_2_O_2_

**Fig 1:**
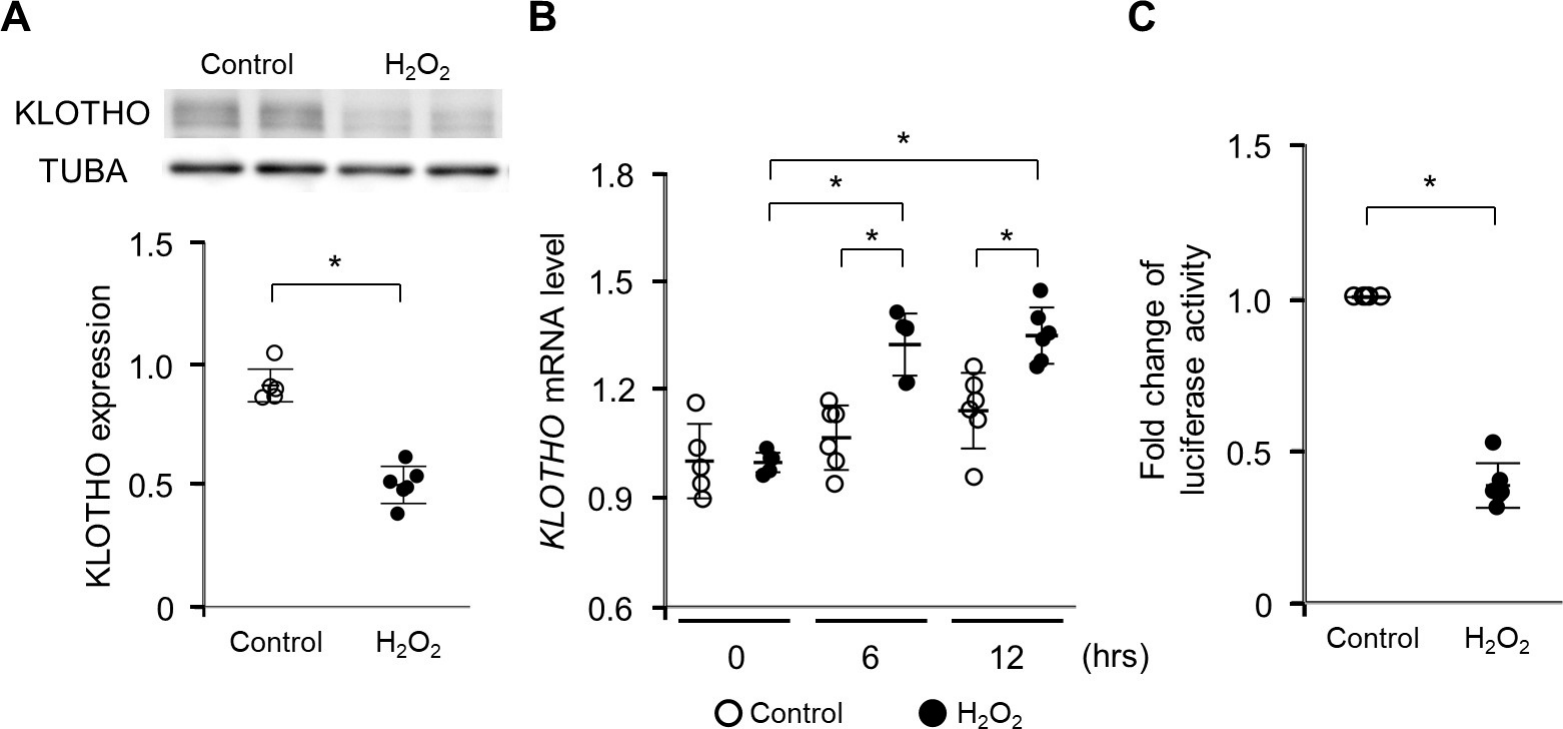
H_2_O_2_ suppresses KLOTHO expression in HK-2 cells at the level of translation. (A) Protein expression of KLOTHO in HK-2 cells treated with 100 μM H_2_O_2_ for 24 hours (hrs). Band intensities were analyzed and normalized against levels of α-TUBULIN (TUBA) using densitometry. (B) Expression of *KLOTHO* mRNA in HK-2 cells treated with 100 μM H_2_O_2_ for the indicated times. (C) Luciferase activities in HK-2 cells transfected with pMirTarget Vector harboring human *KLOTHO* 3′-UTR with or without 100 μM H_2_O_2_ treatment for 12 hrs. Luciferase activity was normalized against protein amount. Values represent individual measurements and the mean ± SD. Data were analyzed using the Mann-Whitney *U*-test or the Mann-Whitney *U*-test with Bonferroni correction. *P < 0.05, n = 6.

We carried out *in vitro* experiments to investigate the underlying mechanism by which oxidative stress regulates *KLOTHO* gene expression in HK-2 cells. First, we examined the mRNA and protein levels in HK-2 cells with or without H_2_O_2_ stimulation. Protein levels of KLOTHO decreased in HK-2 cells with H_2_O_2_ stimulation, whereas *KLOTHO* mRNA levels were induced at 6 hrs and 12 hrs compared with controls (Fig 1A and 1B).

Next, we used a luciferase reporter system to investigate the effect of H_2_O_2_ on the translation of *KLOTHO* mRNA in HK-2 cells. A reporter plasmid harboring the 3′-UTR of *KLOTHO* mRNA was used to analyze whether the expression of KLOTHO is mediated by its 3′-UTR. The activity of the luciferase reporter harboring the *KLOTHO* mRNA 3′-UTR was significantly reduced by H_2_O_2_ stimulation in HK-2 cells (Fig 1C).

### miR-200c and miR-21 are upregulated by H_2_O_2_

**Fig 2:**
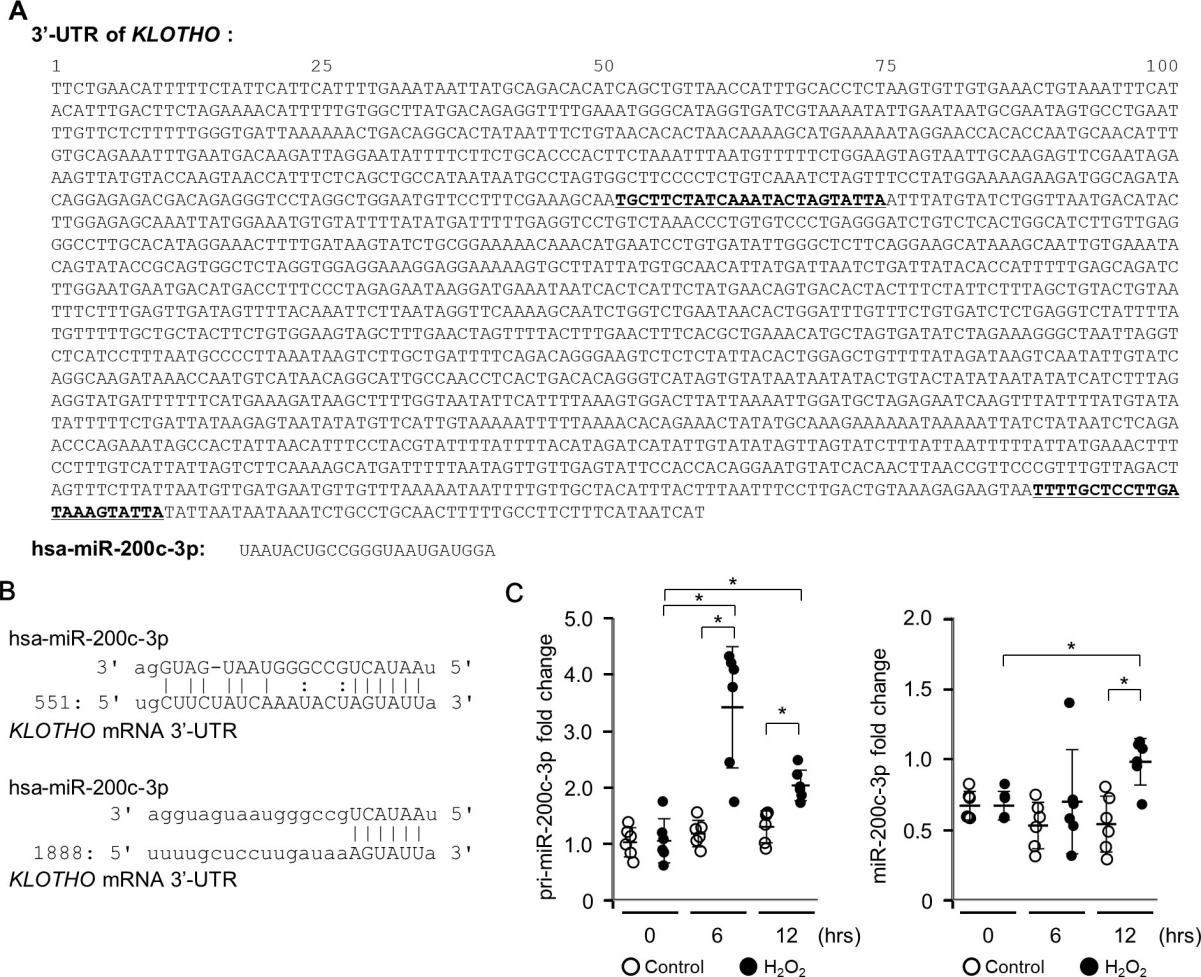
miR-200c is complementary to the human *KLOTHO* mRNA 3′-UTR and is upregulated by H_2_O_2_ exposure. (A) Putative miR-200c binding sites (underlined sequence) in the *KLOTHO* 3′-UTR sequence predicted by an online algorithm (www.microrna.org). (B) Predicted target sites of miR-200c in the *KLOTHO* mRNA 3′-UTR. There are two possible binding sites. (C) q-PCR analysis of pri-miR-200c and miR-200c expression in HK-2 cells cultured with or without 100 μM H_2_O_2_ at the indicated time points. U6 snRNA was used for normalization. Values represent individual measurements and the mean ± SD. Data were analyzed using the Mann-Whitney *U*-test with Bonferroni correction. *P < 0.05, n = 6.

We used the online prediction tool, microRNA.org [39], to assess the potential binding of miR-200c to the 3′-UTR of *KLOTHO* mRNA. There are two possible miR-200c binding sites in the *KLOTHO* 3′-UTR (Fig 2A and 2B). Base pairing between the *KLOTHO* 3′-UTR and miR-200c is shown (Fig 2B). Quantification of the expression of pri-miR-200c and miR-200c in HK-2 cells showed they were significantly induced by H_2_O_2_ stimulation (Fig 2C). We also found that microRNA-21 (miR-21), another miRNA with a predicted binding sequence in the 3′-UTR of *KLOTHO* mRNA, was upregulated by H_2_O_2_ stimulation (Part A in S1 Fig).

### miR-200c inhibits KLOTHO expression

**Fig 3:**
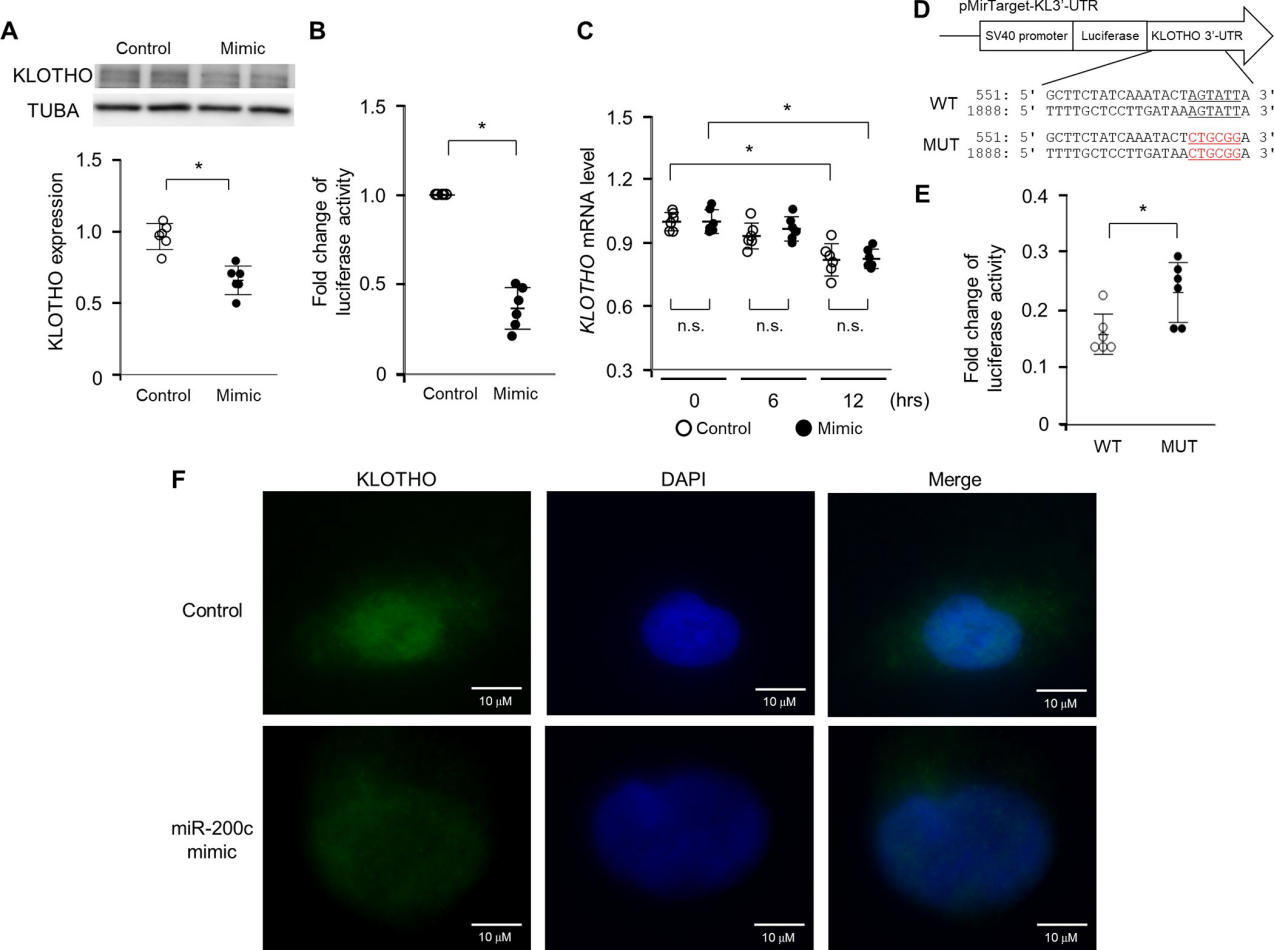
miR-200c decreases KLOTHO expression in HK-2 cells by translational repression. (A) KLOTHO protein expression in HK-2 cells 24 hrs after transfection with 25 nM mimic control or miR-200c mimic. Cells were cultured for another 48 hrs without mimic control or miR-200c mimic before sampling. Band intensities were analyzed and normalized against TUBA using densitometry. (B) A *KLOTHO* 3′-UTR reporter plasmid in combination with 50 nM mimic control or miR-200c mimic was transfected into HK-2 cells for 4 hrs. After a medium change, HK-2 cells were cultured for another 12 hrs before sampling. Luciferase activity was normalized against protein amount. (C) HK-2 cells were transfected with 50 nM mimic control or miR-200c mimic for 4 hrs and cultured for another 24 hrs before harvesting total RNA. *KLOTHO* mRNA levels were evaluated by q-PCR. (D) Mutations were introduced into the 3′-UTR of *KLOHO* mRNA as indicated. (E) The effect of 100 nM miR-200c mimic on the reporter activity of wild type (WT, pMirTarget-KL3′-UTR-WT) and mutant (MUT, pMirTarget-KL3′-UTR-MUT) plasmids in HK-2 cells was measured by luciferase assay. (F) HK-2 cells were stained with anti-KLOTHO antibody and Alexa Fluor 488-labeled goat anti-rabbit IgG. KLOTHO protein was evaluated under fluorescence microscopy. Scale bar = 10 μm. Values represent individual measurements and the mean ± SD. Data were analyzed using the Mann-Whitney *U*-test or the Mann-Whitney *U*-test with Bonferroni correction. *P < 0.05, n = 6. n.s.; not significant.

To examine the inhibitory effect of miR-200c on the metabolism of *KLOTHO* mRNA, we compared protein levels, luciferase activity and mRNA levels in HK-2 cells with or without the transfection of miR-200c. The transfection of miR-200c suppressed protein levels of KLOTHO in HK-2 cells (Fig 3A). Consistent with these results, co-transfection of miR-200c with the *KLOTHO* 3′-UTR reporter plasmid dampened luciferase activity (Fig 3B). Moreover, we examined the mRNA expression of *KLOTHO* in HK-2 cells to determine the effect of miR-200c on *KLOTHO* mRNA metabolism. The expression of *KLOTHO* mRNA was not significantly different between HK-2 cells transfected with miR-200c or control RNA (Fig 3C). Bioinformatics analysis indicated the presence of two potential binding sites of miR-200c in the *KLOTHO* 3′-UTR. To determine whether these sites were actual targets of miR-200c, we mutated both sites (Fig 3D) and examined plasmid luciferase activity. Mutations of these sites restored luciferase activity (Fig 3E). Immunofluorescent assay revealed that the miR-200c mimic reduced KLOTHO expression, and the expression of KLOTHO was observed in the cytosol and nuclei of HK-2 cells (Fig 3F).

**Fig 4:**
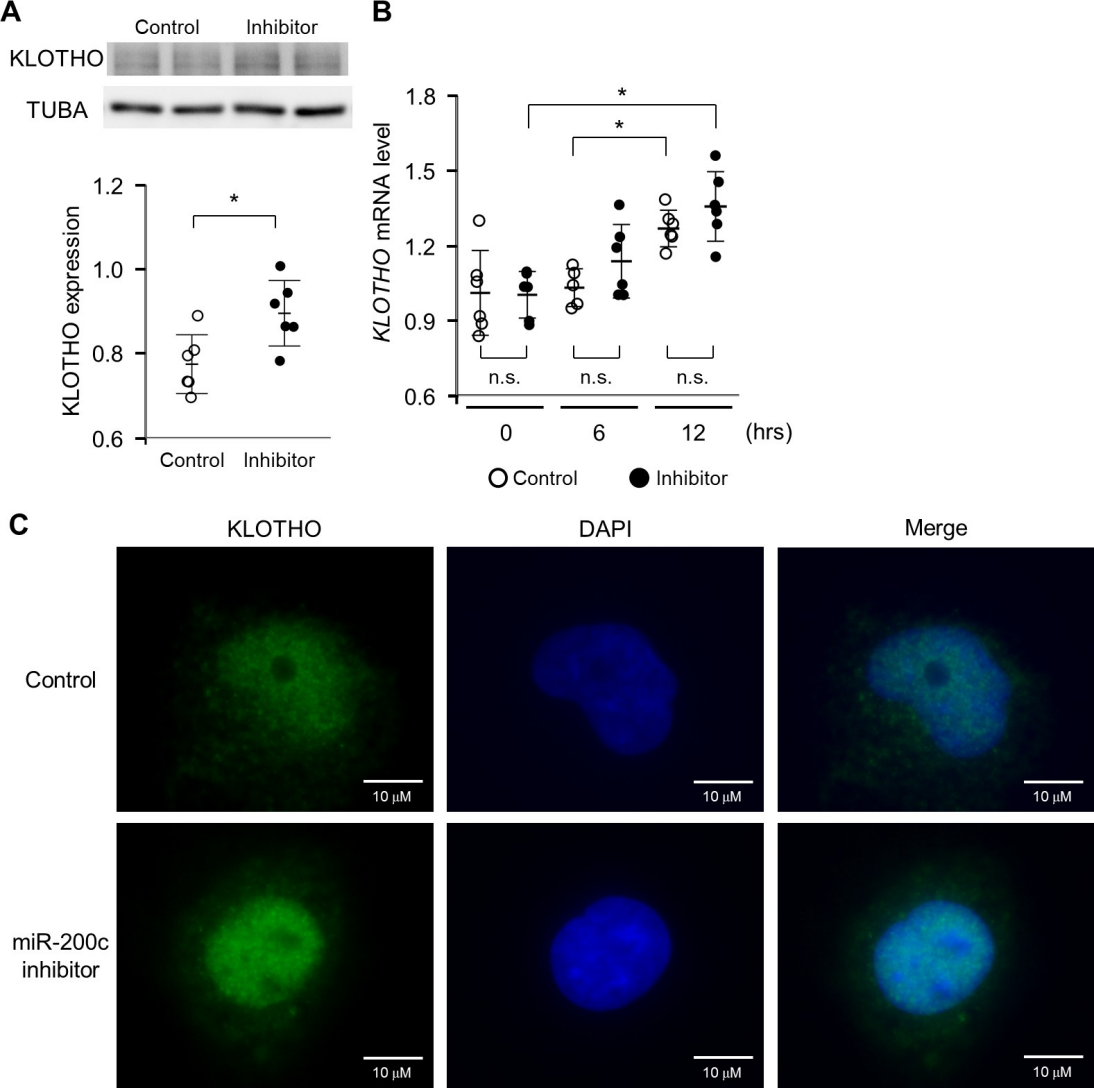
KLOTHO protein is preserved by inhibiting miR-200c in HK-2 cells. (A) The effect of a miR-200c inhibitor on KLOTHO protein expression in HK-2 cells treated with H_2_O_2_. KLOTHO protein expression in HK-2 cells treated with 100 μM H_2_O_2_ for 24 hrs after the transfection of inhibitor control (25 nM) or miR-200c inhibitor (25 nM) for 4 hrs. Band intensities were analyzed and normalized against TUBA using densitometry. *P < 0.05, n = 6 (B) The effect of a miR-200c inhibitor on *KLOTHO* mRNA expression in HK-2 cells treated with H_2_O_2_. HK-2 cells were transfected with inhibitor control (25 nM) or miR-200c inhibitor (25 nM) and 12 hrs later were treated with 100 μM H_2_O_2_. *KLOTHO* mRNA was detected by q-PCR. (C) HK-2 cells were stained with anti-KLOTHO antibody and Alexa Fluor 488-labeled goat anti-rabbit IgG. KLOTHO protein was evaluated under fluorescence microscopy. Scale bar = 10 μm. Values represent individual measurements and the mean ± SD. Data were analyzed using the Mann-Whitney *U*-test or the Mann-Whitney *U*-test with Bonferroni correction. *P < 0.05, n = 6. n.s.; not significant.

We further examined the effect of miR-200c on KLOTHO expression in H_2_O_2_-stimulated HK-2 cells with or without transfection of the miR-200c inhibitor. As shown in Fig 4A, the miR-200c inhibitor upregulated the protein level of KLOTHO compared with control RNA. The expression level of *KLOTHO* mRNA was not significantly different between HK-2 cells transfected with miR-200c inhibitor or control RNA (Fig 4B), although *KLOTHO* mRNA was upregulated by H_2_O_2_ stimulation compared with control transfected HK-2 cells without exposure to H_2_O_2_ (S2 Fig). By immunofluorescent assay, miR-200c inhibitor was shown to retain KLOTHO expression (Fig 4C).

The H_2_O_2_-induced miR-21 also has a putative binding site in the *KLOTHO* mRNA 3′-UTR; however, the miR-21 inhibitor did not improve KLOTHO protein expression (Part B in S1 Fig). Paraquat, another oxidative stress inducer, reduced KLOTHO expression (S3 Fig).

### KLOTHO expression is inversely correlated with levels of oxidative stress markers and miR-200c in human kidney specimens

**Fig 5:**
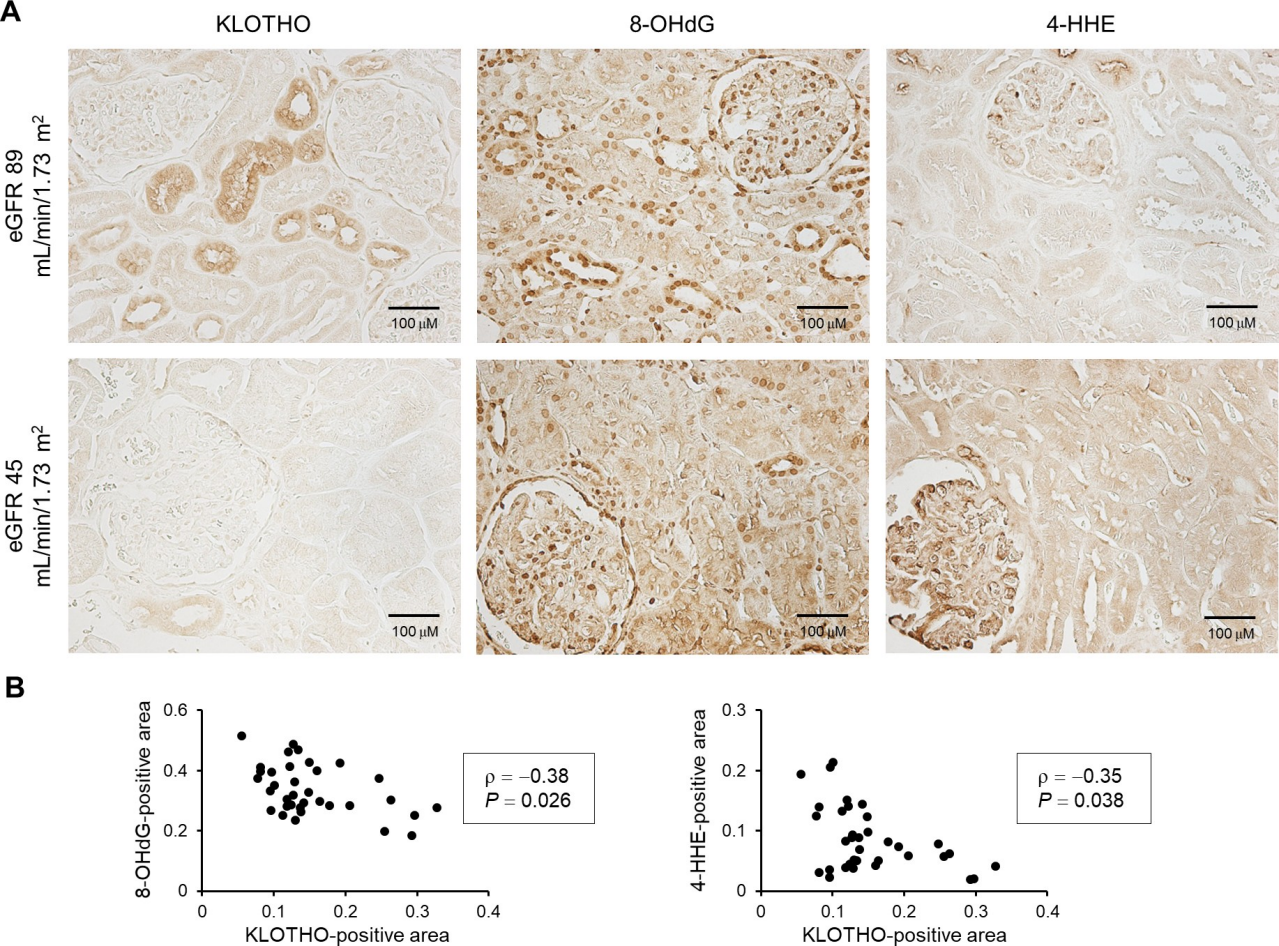
KLOTHO expression inversely correlates with oxidative stress markers in kidney biopsy specimens from IgA nephropathy patients. (A) Representative images of KLOTHO and oxidative stress markers (8-OHdG and 4-HHE) in patients with immunoglobulin A nephropathy. The levels of 8-OHdG and 4-HHE were higher, and those of KLOTHO were lower, in kidney specimens from patients with reduced eGFR compared with patients with conserved eGFR. Scale bar = 100 μm. (B) KLOTHO levels are inversely correlated with 8-OHdG (ρ = −0.38, *P* = 0.026) and 4-HHE (ρ = −0.35, *P* = 0.038) levels. Correlations were calculated using Spearman’s rank correlation coefficient. n = 35.

We examined the degree of oxidative stress in renal biopsy samples obtained from patients with IgA nephropathy (n = 35) by immunohistochemical staining. Details of the clinical characteristics are shown in S1 Table. Consistent with a previous report [38], the expression of oxidative stress markers, 8-OHdG and 4-HHE, were clearly detected in all samples. To evaluate the link between oxidative stress and KLOTHO expression in human kidneys with IgA nephropathy, we performed immunohistochemical staining for KLOTHO. As shown in Fig 5A, KLOTHO was detected in distal renal tubules and was inversely correlated with levels of 8-OHdG (ρ = −0.38, *P* = 0.026) and 4-HHE (ρ = −0.35, *P* = 0.038) (Fig 5B).

**Fig 6:**
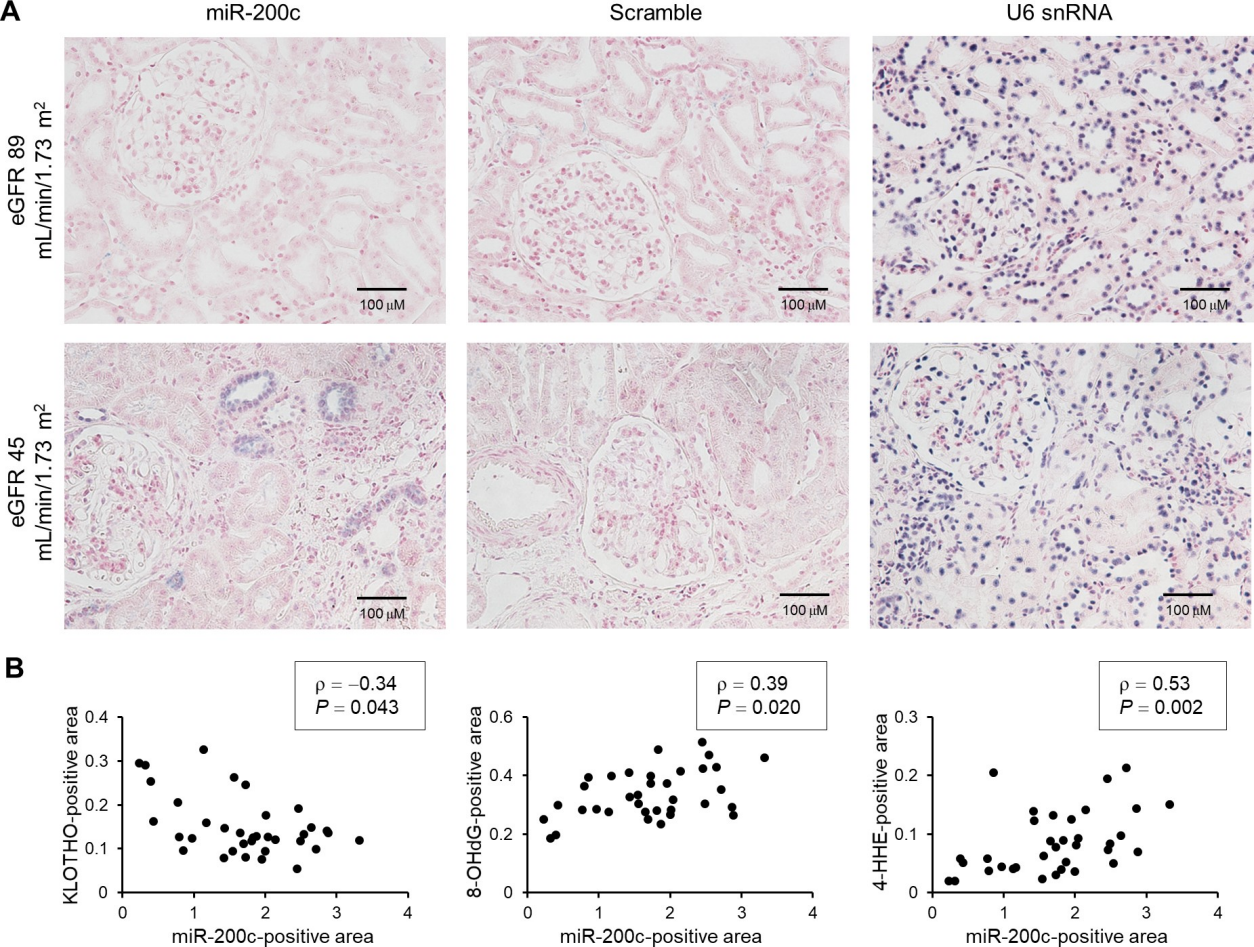
miR-200c expression inversely correlates with KLOTHO expression, and positively correlates with oxidative stress marker levels in kidney biopsy specimens from IgA nephropathy patients. (A) Representative images of miR-200c in the kidneys of patients with IgA nephropathy detected by *in situ* hybridization. Scrambled and U6 snRNA probes were used as negative and positive controls, respectively. Scale bar = 100 μm. (B) miR-200c levels are inversely correlated with KLOTHO levels (ρ = −0.34, *P* = 0.043), but positively correlated with 8-OHdG (ρ = 0.39, *P* = 0.020) and 4-HHE (ρ = 0.53, *P* = 0.002) levels. Correlations were calculated using Spearman’s rank correlation coefficient. n = 35.

We also examined miR-200c expression in the same series of specimens using ISH. miR-200c was detected in distal renal tubules (Fig 6A). Consistent with our *in vitro* examination, miR-200c expression was inversely correlated with KLOTHO expression (ρ = −0.34, *P* = 0.043), whereas it was positively correlated with levels of 8-OHdG (ρ = 0.39, *P* = 0.020) and 4-HHE (ρ = 0.53, *P* = 0.002) (Fig 6B).

## Discussion

In this study, we show that H_2_O_2_ suppressed KLOTHO expression in HK-2 cells. We also show that H_2_O_2_ induced the expression of miR-200c, which has two putative binding sites in the 3′-UTR of *KLOTHO* mRNA. Our *KLOTHO* 3′-UTR reporter assay indicated that miR-200c downregulates KLOTHO expression. In renal biopsy specimens of patients with IgA nephropathy, miR-200c was mainly detected by ISH in distal tubules, where KLOTHO is also expressed. Moreover, the KLOTHO immunostained area was inversely correlated with areas positive for oxidative stress markers and miR-200c. Importantly, another miRNA candidate that we expected to regulate KLOTHO expression, miR-21, did not affect KLOTHO expression. These data indicate that oxidative stress reduces KLOTHO expression through the induction of miR-200c.

miRNA binds to the 3′-UTR of a target mRNA to suppress target gene expression by inhibiting translation or mRNA degradation [33]. In the present study, H_2_O_2_ inhibited KLOTHO protein expression without reducing *KLOTHO* mRNA levels, indicating that miR-200c suppresses KLOTHO expression at the mRNA level, not at the transcriptional level. Indeed, endonucleolytic cleavage of mRNA occurs only when the sequence of the miRNA is completely complementary with that of the target gene, and this is rare in mammals [40,41]. As shown in Fig 2B, the miR-200c sequence and the putative binding sites in the 3′-UTR of *KLOTHO* mRNA were not perfectly matched in humans. We also provide evidence that the transfection of a miR-200c mimic reduced KLOTHO expression, and that luciferase activity was decreased without any reduction in *KLOTHO* mRNA levels. These findings suggest that miR-200c inhibits KLOTHO expression through translational repression, but not by the degradation of *KLOTHO* mRNA.

A number of studies have described the involvement of oxidative stress in the development of various kidney diseases, such as diabetic kidney disease (DKD) [42], and acute kidney injury (AKI) [43,44]. However, in the BEACON trial (Bardoxolone Methyl Evaluation in Patients with Chronic Kidney Disease and Type 2 Diabetes Mellitus: the Occurrence of Renal Events), antioxidant therapy with bardoxolone methyl increased the risk for cardiovascular disease without a beneficial effect on the incidence of end-stage kidney disease in patients with DKD [45]. A possible explanation is that the oxidative response *in vivo* is not always detrimental and may be physiologically important. Therefore, the systemic inhibition of oxidative stress may lead to adverse effects. However, KLOTHO overexpression exhibited a protective effect in various rodent models of renal diseases as well as heart diseases [23,46–48]. The current data suggest that KLOTHO downregulation induced by oxidative stress is an attractive therapeutic strategy.

Renal fibrosis is the most common pathological feature of CKD regardless of the underlying disease [49]. During the development of renal fibrosis, a major source of extracellular matrix (ECM) proteins results from the transformation of resident fibroblast cells into myofibroblasts, while epithelial-mesenchymal transition (EMT) accounts for 10% of ECM proteins in this process [50]. miR-200a prevents EMT, leading to protection from renal fibrosis [51,52], while miR-29 inhibits renal fibrosis through the prevention of ECM deposition [53–56]. These miRNAs can be regarded as beneficial for kidneys; however, several miRNAs might have detrimental effects on kidneys. For example, miR-21 [57,58], miR-192 [59–61], and miR-433 [62], exacerbated renal fibrosis in mice. Both miR-339 and miR-556 decreased the expression of KLOTHO *in vitro* [63], and in this study we show that oxidative stress-induced miR-200c was involved in repressing KLOTHO protein expression, because transfection of a miR-200c inhibitor maintained KLOTHO expression. KLOTHO deficiency caused renal fibrosis, whereas the overexpression or injection of KLOTHO ameliorated it [23,24,27]. Combined, these findings indicate that the inhibition of miR-200c exhibits a beneficial effect in tissues that express KLOTHO protein.

The inhibition of miR-200c induced the expression of zinc finger E-box-binding homeobox (ZEB) 1 and ZEB2, resulting in the promotion of EMT through a reduction of E-cadherin in cells that do not express KLOTHO [64,65]. In contrast, KLOTHO protein confers the ability to prevent EMT by various mechanisms, such as PI3K/Akt.GSKβ3/Snail signaling [66], Wnt/β-catenin signaling [67], and TGF-β1 signaling [24]. Thus, the effect of miR-200c on renal fibrosis remains controversial despite our assumption that the inhibition of miR-200c may show beneficial effects against renal fibrosis. It should, therefore, be investigated in an animal model of renal fibrosis. However, the *KLOTHO* 3′-UTR sequence is different between humans and rodents, raising the possibility that another miRNA may influence KLOTHO expression in mice or rats. Moreover, although previous studies have reported that H_2_O_2_ induced the activation of mitogen-activated protein kinases [68,69], the inhibition of these signaling pathways did not improve KLOTHO expression (S4 Fig). Major limitations of this study are that we did not assess the actual effect of miR-200c on KLOTHO expression *in vivo*, and that we could not identify the transcriptional factor responsible for miR-200c-mediated KLOTHO downregulation.

Although immunohistochemistry indicated that KLOTHO was expressed mainly in the cytoplasm of distal tubular cells and not the nucleus, immunofluorescent staining of HK-2 cells revealed that, in addition to the cytosol, KLOTHO was present in the nucleus. HK-2 cells are mainly derived from proximal tubular cells, suggesting that the cellular localization of KLOTHO might be different between cell types. Previous studies reported that KLOTHO exists as secreted, transmembrane and intracellular forms [18,70,71], and that KLOTHO expression is observed at the peripheral portion of the nucleus and the nucleolus in choroid plexus cells and cerebellar Purkinje cells [72]. These findings indicate that the localization of KLOTHO in HK-2 cells is similar to that in brain cells. However, miRNAs were reportedly localized at all major cellular organelles [73]. In this study, we obtained consistent data from our *in vitro* study and immunohistochemistry on human biopsy samples that oxidative stress decreased KLOTHO expression even though its localization was different in these experiments. The resulting data suggest that oxidative stress-induced miR-200c plays an important role in the downregulation of KLOTHO in proximal and distal tubular cells.

**Fig 7:**
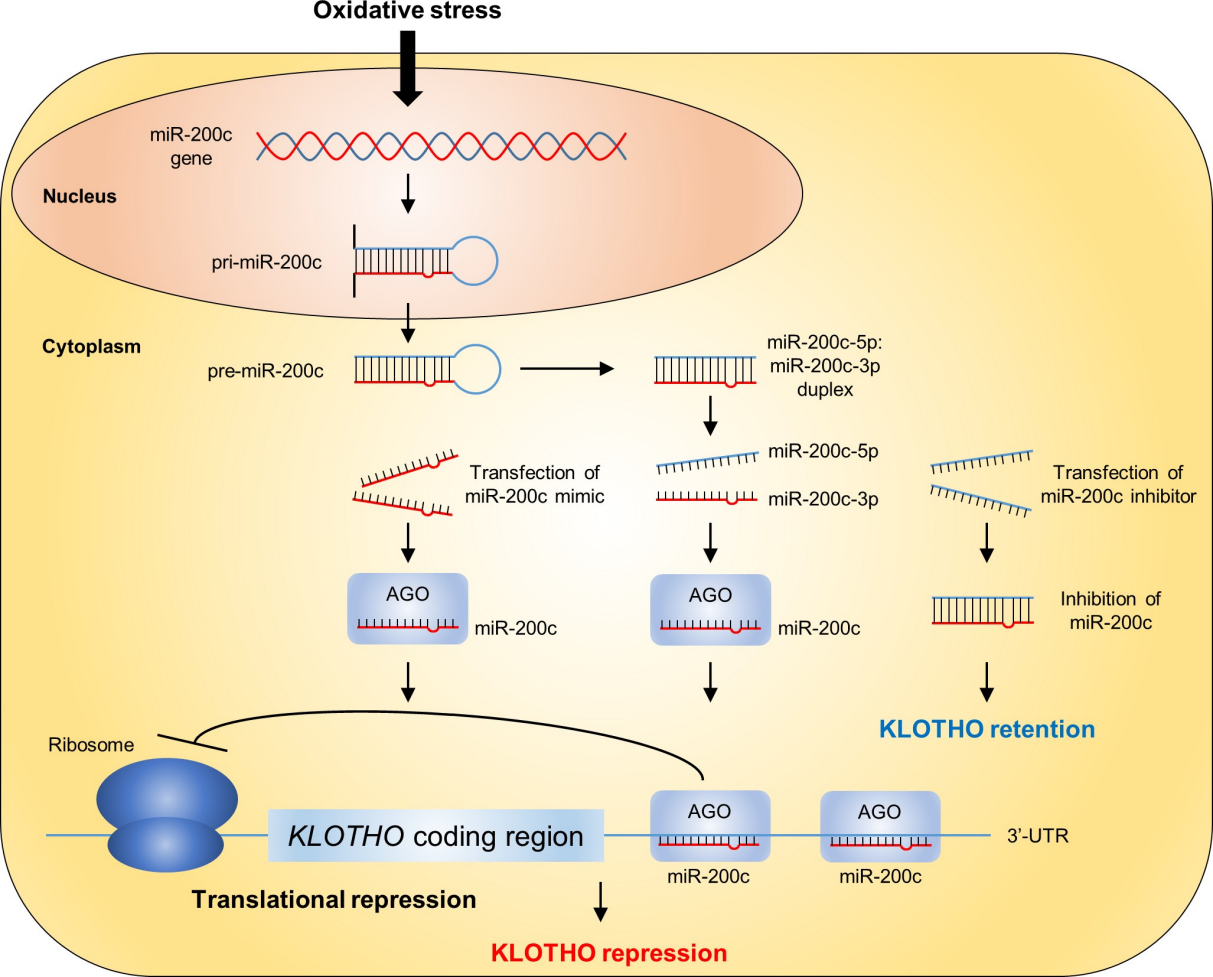
Summary of the results. In HK-2 cells, oxidative stress induced pri-miR-200c and miR-200c, a miRNA that is complementary with the 3′-UTR of *KLOTHO* mRNA at two different sites. miR-200c binds to the *KLOTHO* mRNA 3′-UTR with Argonaute protein (AGO) to suppress the expression of KLOTHO by inhibiting translation. Transfection of a miR-200c mimic reduced KLOTHO expression without a reduction in *KLOTHO* mRNA levels and transfection of a miR-200c inhibitor maintained KLOTHO expression.

In summary, we show that H_2_O_2_ suppresses KLOTHO expression without a reduction in *KLOTHO* mRNA levels. The luciferase activity of a *KLOTHO* 3′-UTR reporter was decreased in response to H_2_O_2_ stimulation, indicating that an H_2_O_2_-induced miRNA regulates KLOTHO expression. A candidate miRNA is miR-200c, which has two possible binding sites in the *KLOTHO* 3′-UTR. Transfection of a miR-200c mimic decreased KLOTHO expression, whereas transfection of a miR-200c inhibitor maintained KLOTHO expression (Fig 7). In human renal biopsy samples, the levels of oxidative stress markers, such as 8-OHdG and 4HHE, were correlated with miR-200c and KLOTHO expression. These findings suggest that oxidative stress suppresses KLOTHO expression through the induction of miR-200c.

## Supporting information

S1 Fig miR-21 is upregulated by H_2_O_2_ exposure, but does not alter KLOTHO expression in HK-2 cells. (A) q-PCR analysis of pri-miR-21 and miR-21 expression in HK-2 cells cultured with or without 100 μM H_2_O_2_ at the indicated time points. U6 snRNA was used for normalization. (B) KLOTHO protein expression in HK-2 cells treated with 100 μM H_2_O_2_ for 24 hrs after the transfection of an inhibitor control (50 nM) or miR-21 inhibitor (50 nM) for 24 hrs. Band intensities were analyzed and normalized against TUBA using densitometry. *P < 0.05, n = 6. Values represent individual measurements and the mean ± SD. Data were analyzed using the Mann-Whitney *U*-test or the Mann-Whitney *U*-test with Bonferroni correction. n.s.; not significant.
(TIF)

S2 Fig Upregulation of *KLOTHO* mRNA levels by H_2_O_2_ stimulation is conserved in miR-200c inhibitor transfected HK-2 cells. The effect of H_2_O_2_ stimulation on *KLOTHO* mRNA expression in HK-2 cells transfected with miR-200c inhibitor was investigated. HK-2 cells were transfected with inhibitor control (25 nM) or miR-200c inhibitor (25 nM) and 12 hrs later they were treated with 100 μM H_2_O_2_. *KLOTHO* mRNA was detected by q-PCR. *P < 0.05, n = 6. Values represent individual measurements and the mean ± SD. Data were analyzed using the Mann-Whitney *U*-test or the Mann-Whitney *U*-test with Bonferroni correction.
(TIF)

S3 Fig Paraquat suppresses KLOTHO expression in HK-2 cells. KLOTHO protein expression in HK-2 cells treated with Paraquat for 24 hrs.
(TIF)

S4 Fig Inhibition of the MAP kinase pathway does not restore KLOTHO suppression by H_2_O_2_ in HK-2 cells. (A, C, E) KLOTHO protein expression in HK-2 cells treated with 100 μM H_2_O_2_ for 24 hrs after the transfection of siRNAs (si-ERK, si-JNK and si-p38) or negative control siRNA (25 nM) for 24 hrs. Band intensities were analyzed and normalized against TUBA using densitometry. (B, D, E) MAP kinase expression in HK-2 cells treated with siRNAs. MAP; Mitogen-activated Protein, ERK; Extracellular Signal-regulated Kinase, JNK; c-Jun N-terminal Kinase.
(TIF)

S5 Fig Full length western blots for Figs 1A, 3A and 4A. (TIF)

S1 Table Clinical characteristics related to renal function of IgA nephropathy patients. (TIF)
