# Supplementary figures and images for: microRNA-200c regulates KLOTHO expression in human kidney cells under oxidative stress
Source: PLoS One. 2019 Jun 14;14(6):e0218468. doi: 10.1371/journal.pone.0218468 (PMC6568409; doi:10.1371/journal.pone.0218468)

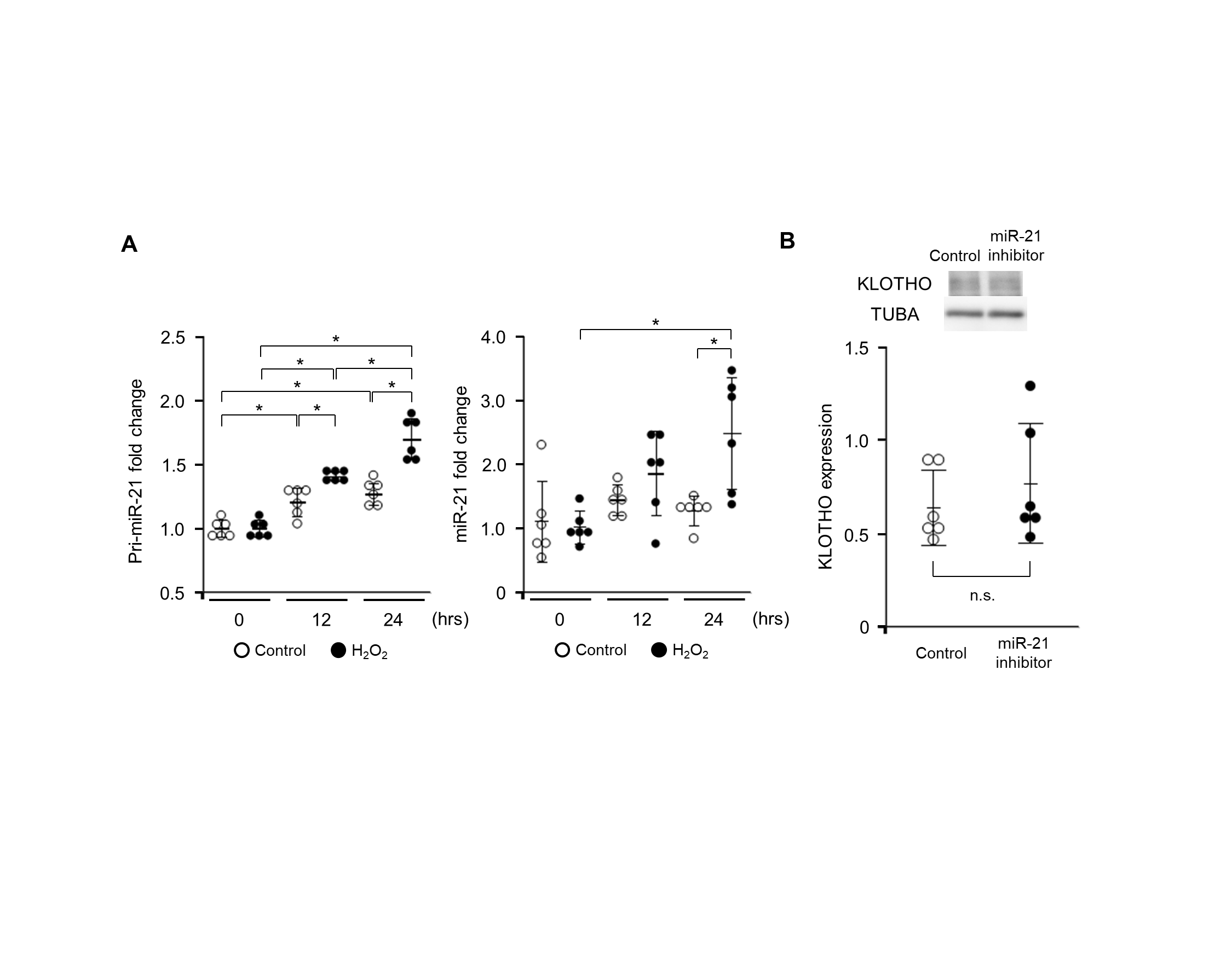

Supplement: S1 Fig — (A) q-PCR analysis of pri-miR-21 and miR-21 expression in HK-2 cells cultured with or without 100 μM H2O2 at the indicated time points. U6 snRNA was used for normalization. (B) KLOTHO protein expression in HK-2 cells treated with 100 μM H2O2 for 24 hrs after the transfection of an inhibitor control (50 nM) or miR-21 inhibitor (50 nM) for 24 hrs. Band intensities were analyzed and normalized against TUBA using densitometry. *P < 0.05, n = 6. Values represent individual measurements and the mean ± SD. Data were analyzed using the Mann-Whitney U-test or the Mann-Whitney U-test with Bonferroni correction. n.s.; not significant. (TIF) [file pone.0218468.s001.TIF]

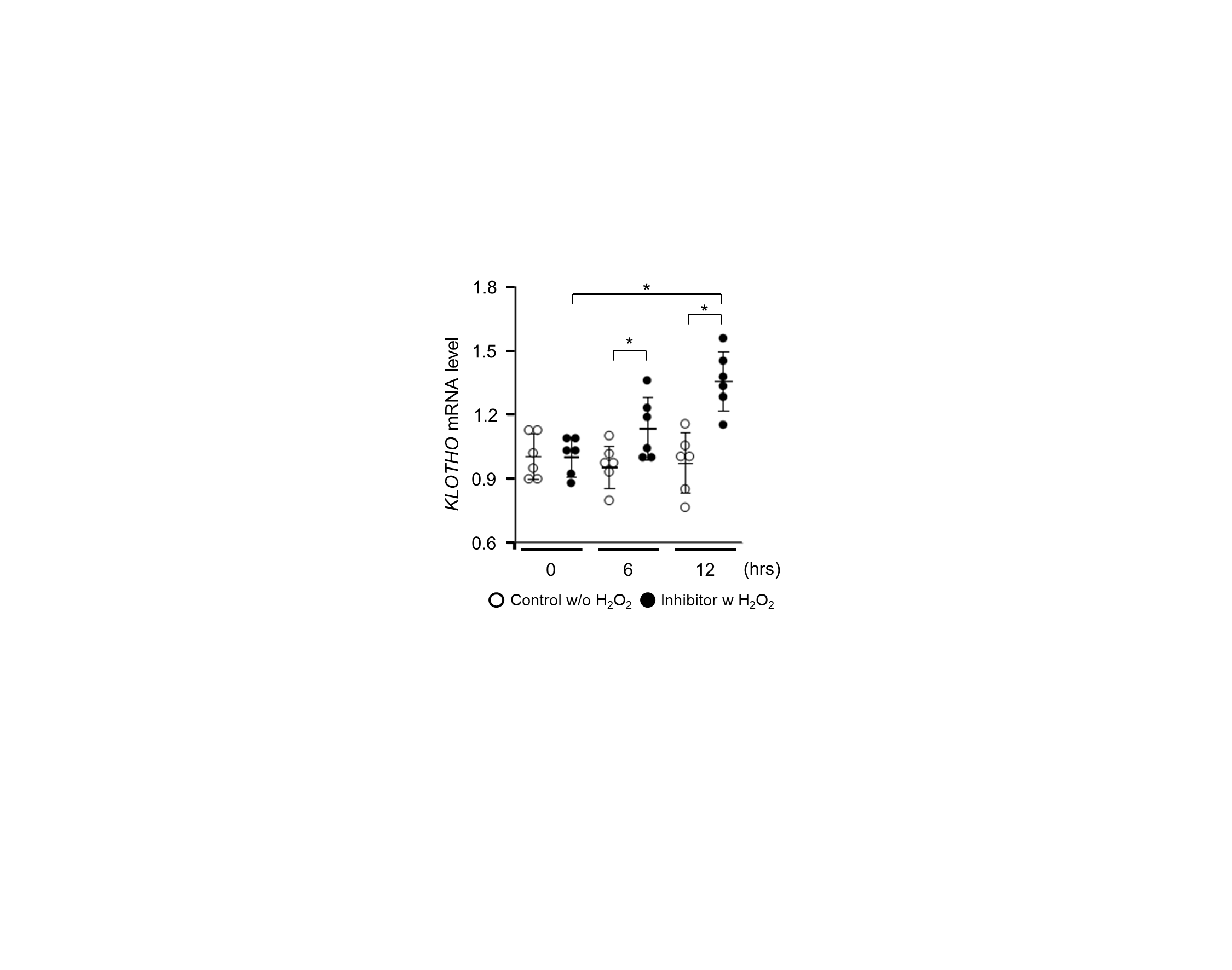

Supplement: S2 Fig — The effect of H2O2 stimulation on KLOTHO mRNA expression in HK-2 cells transfected with miR-200c inhibitor was investigated. HK-2 cells were transfected with inhibitor control (25 nM) or miR-200c inhibitor (25 nM) and 12 hrs later they were treated with 100 μM H2O2. KLOTHO mRNA was detected by q-PCR. *P < 0.05, n = 6. Values represent individual measurements and the mean ± SD. Data were analyzed using the Mann-Whitney U-test or the Mann-Whitney U-test with Bonferroni correction. (TIF) [file pone.0218468.s002.TIF]

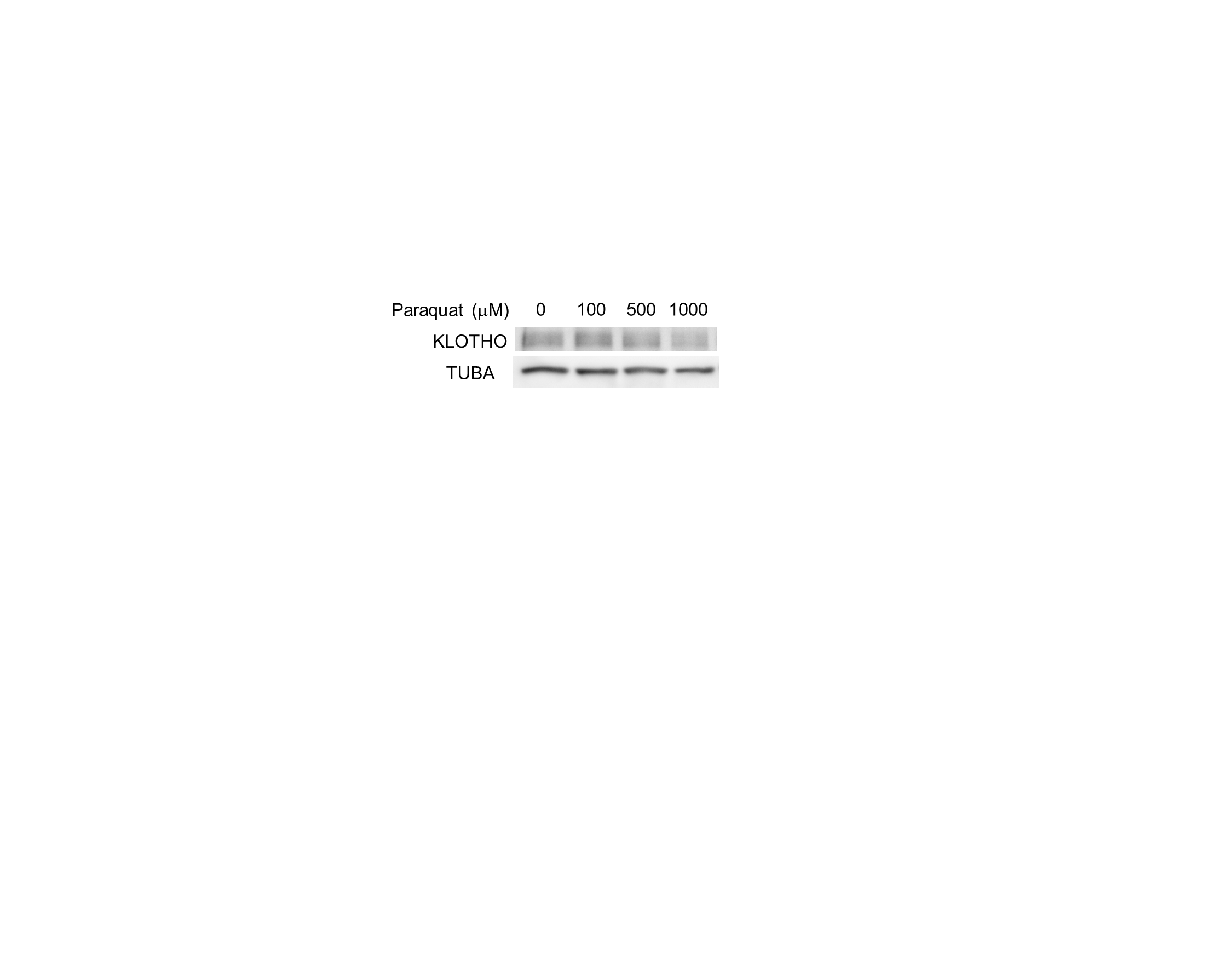

Supplement: S3 Fig — KLOTHO protein expression in HK-2 cells treated with Paraquat for 24 hrs. (TIF) [file pone.0218468.s003.TIF]

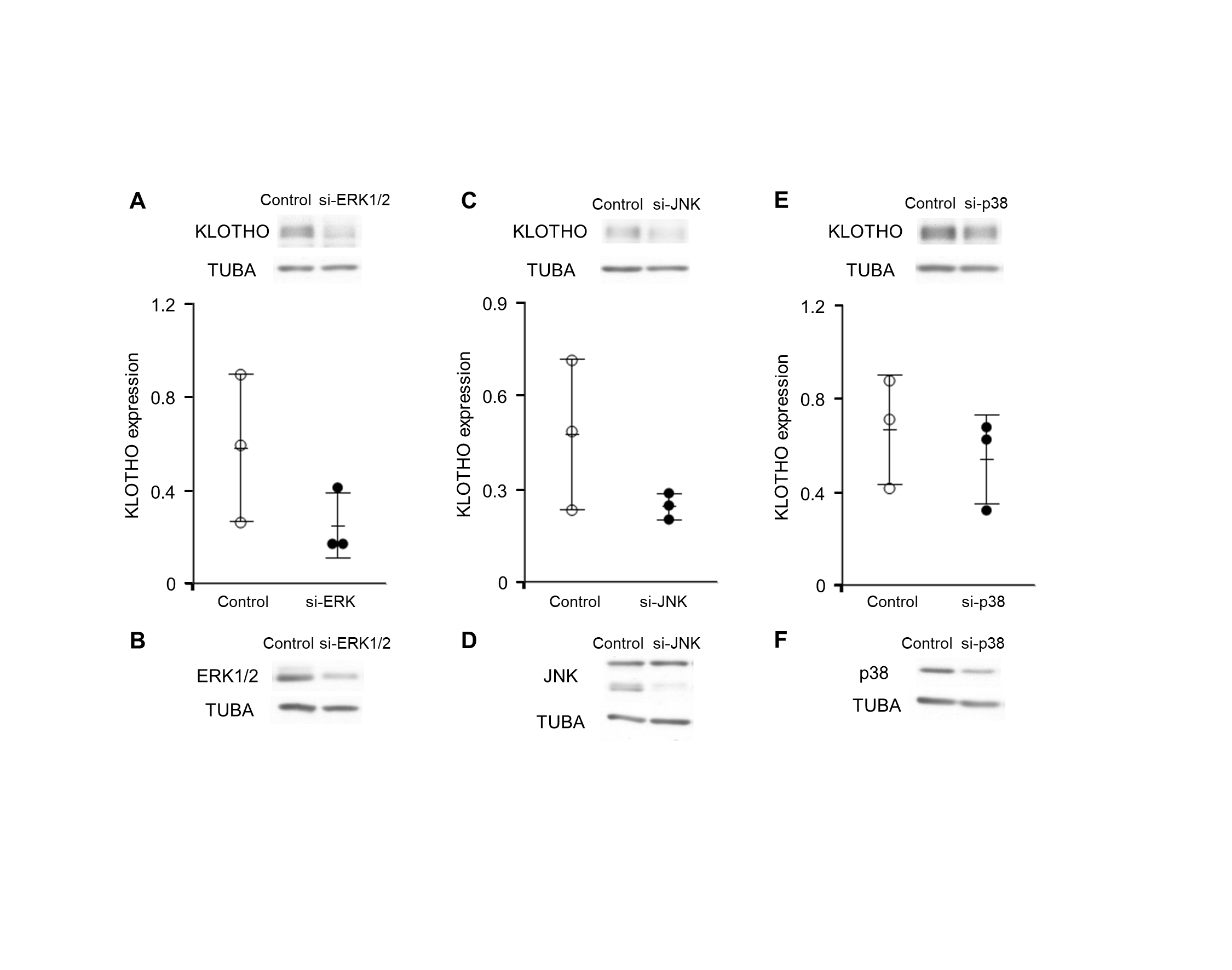

Supplement: S4 Fig — (A, C, E) KLOTHO protein expression in HK-2 cells treated with 100 μM H2O2 for 24 hrs after the transfection of siRNAs (si-ERK, si-JNK and si-p38) or negative control siRNA (25 nM) for 24 hrs. Band intensities were analyzed and normalized against TUBA using densitometry. (B, D, E) MAP kinase expression in HK-2 cells treated with siRNAs. MAP; Mitogen-activated Protein, ERK; Extracellular Signal-regulated Kinase, JNK; c-Jun N-terminal Kinase. (TIF) [file pone.0218468.s004.TIF]

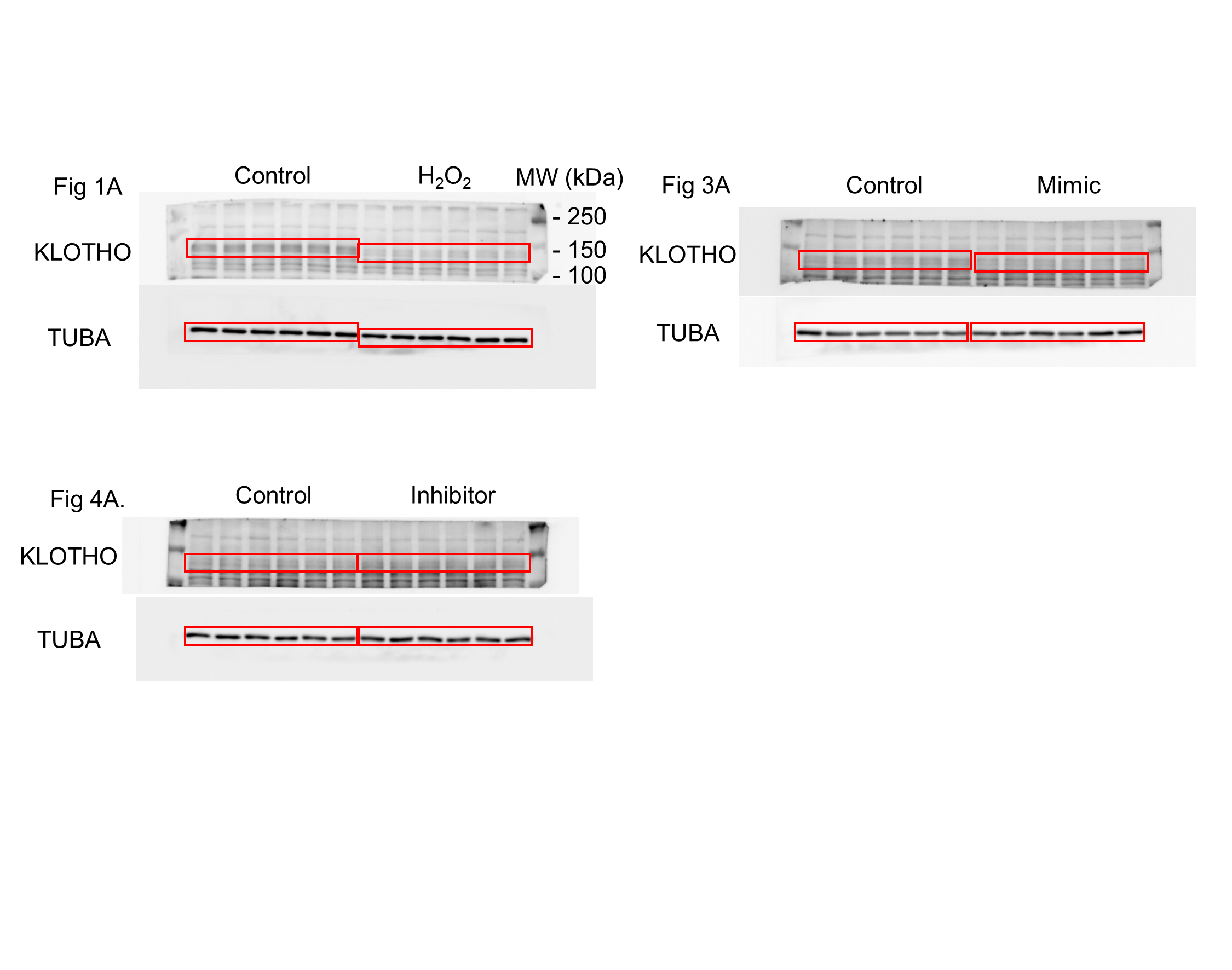

Supplement: S5 Fig — (TIF) [file pone.0218468.s005.TIF]

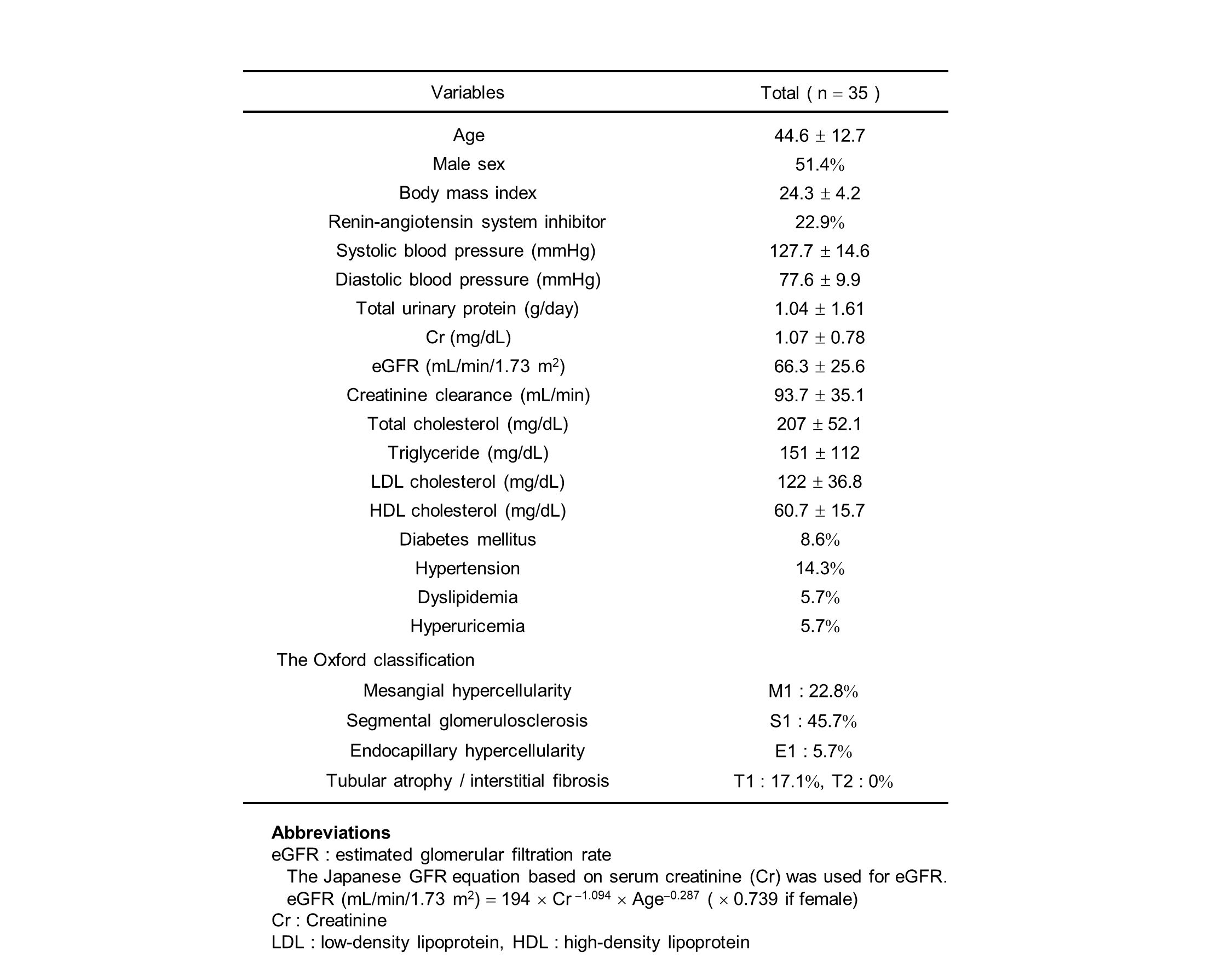

Supplement: S1 Table — (TIF) [file pone.0218468.s006.TIF]
